# Twin-to-twin transfusion syndrome neurodevelopmental follow-up study (neurodevelopmental outcomes for children whose twin-to-twin transfusion syndrome was treated with placental laser photocoagulation)

**DOI:** 10.1186/s12887-018-1230-8

**Published:** 2018-08-01

**Authors:** Christie Bolch, Michael Fahey, Dinah Reddihough, Katrina Williams, Susan Reid, Angela Guzys, Stephen Cole, Andrew Edwards, Alison Fung, Ryan Hodges, Ricardo Palma-Dias, Mark Teoh, Susan Walker

**Affiliations:** 1 Developmental Disability and Rehabilitation Research, Murdoch Children’s Research Institute, Melbourne, Australia; 2 Neurodevelopment and Disability, The Royal Children’s Hospital, Melbourne, Australia; 3 Department of Paediatrics, Monash Health, Clayton, Australia; 4 Department of Paediatrics, Monash University, Clayton, Australia; 5 Department of Paediatrics, The University of Melbourne, Melbourne, Australia; 6 Department of Maternity Services, Royal Women’s Hospital, Melbourne, Australia; 7 Hudson Institute of Medical Research, Monash University, Clayton, VIC Australia; 8 Department of Perinatal Medicine, Mercy Hospital for Women, Heidelberg, VIC Australia; 9 Women’s & Newborn Program, Monash Health, Clayton, VIC Australia; 10 The Ritchie Centre, Hudson Institute of Medical Research, Melbourne, Australia; 11 Ultrasound Services, Royal Women’s Hospital, Parkville, VIC Australia; 12 Pregnancy Research Centre, Department of Maternal-Fetal Medicine, Royal Women’s Hospital, Parkville, VIC Australia; 13 Department of Obstetrics and Gynaecology, University of Melbourne, Parkville, VIC Australia; 14 Fetal Diagnostic Unit, Monash Health, Clayton, VIC Australia; 15 Department of Obstetrics and Gynaecology, Mercy Hospital for Women, Heidelberg, Australia; 16 Maternal Fetal Medicine, The University of Melbourne, Melbourne, Australia

**Keywords:** Neurodevelopmental outcomes, Twin-to-twin transfusion syndrome, Laser placental photocoagulation

## Abstract

**Background:**

Twin-to-twin transfusion syndrome (TTTS) is a serious complication of 10–15% of twin or triplet pregnancies in which multiple fetuses share a single placenta. Communicating placental vessels allow one fetus (the donor) to pump blood to the other (the recipient). Mortality rates without intervention are high, approaching 100% in some series, with fetal deaths usually due to cardiac failure. Surgical correction using laser photocoagulation of communicating placental vessels was developed in the 1980s and refined in the 1990s. Since it was introduced in Victoria in 2006, laser surgery has been performed in approximately 120 pregnancies.

Survival of one or more fetuses following laser surgery is currently > 90%, however the neurodevelopmental outcomes for survivors remain incompletely understood. Prior to laser therapy, at least one in five survivors of TTTS had serious adverse neurodevelopmental outcomes (usually cerebral palsy). Current estimates of neurological impairment among survivors following laser surgery vary from 4 to 31% and long-term follow-up data are limited.

**Methods:**

This paper describes the methodology for a retrospective cohort study in which children aged 24 months and over (corrected for prematurity), who were treated with laser placental photocoagulation for TTTS at Monash Health in Victoria, Australia, will undergo comprehensive neurodevelopmental assessment by a multidisciplinary team. Evaluation will include parental completion of pre-assessment questionnaires of social and behavioural development, a standardised medical assessment by a developmental paediatrician or paediatric neurologist, and age-appropriate cognitive and academic, speech and fine and gross motor assessments by psychologists, speech and occupational therapists or physiotherapists. Assessments will be undertaken at the Murdoch Children’s Research Institute/Royal Children’s Hospital, at Monash Health or at another mutually agreed location. Results will be recorded in a secure online database which will facilitate future related research.

**Discussion:**

This will be the first study to report and evaluate neurodevelopmental outcomes following laser surgery for twin-to-twin transfusion syndrome in Victoria, and will inform clinical practice regarding follow-up of children at risk of adverse outcomes.

**Electronic supplementary material:**

The online version of this article (10.1186/s12887-018-1230-8) contains supplementary material, which is available to authorized users.

## Background

### Multiple pregnancy and types of twinning

When a single fertilised egg (zygote) splits into two embryos, a monozygotic twin pregnancy results. This occurs spontaneously in 1 in 250 pregnancies [1]. When a single embryo splits, in ¾ of cases, the developing genetically identical fetuses share a single placenta and are described as “monochorionic”. For monozygotic twins (and more rarely, triplets) who share a placenta, there are significant associated risks, with a 3 and 7-fold increased risk of perinatal illness and death respectively, compared with singletons [2]. They are 9 times more likely to die in utero than identical twins who do not share a placenta, with a perinatal mortality rate of 12% when born at or after 32 weeks [3]. Premature birth is a significant risk for monozygotic multiples, with 63% of twins and all triplets born prior to 37 weeks [4, 5].

### Pathophysiology of twin-to-twin transfusion syndrome

In 10–15% of monozygotic monochorionic pregnancies, blood flows unequally along placental blood vessels known as anastomoses [6]. This condition is known as “twin-to-twin transfusion syndrome” (TTTS). One fetus (identified as the donor) “pumps” blood to the other (the recipient). This situation is extremely hazardous for both.

The donor twin may become severely anaemic; urine output diminishes, growth falters and their amniotic sac empties and shrinks (oligohydramnios). Such a fetus may ultimately become adhered to the membranes (“stuck twin”). Should it survive to birth, his or her lungs may not have developed adequately. At birth, a donor may appear small, malnourished and pale. In contrast, the recipient is at risk of blood volume overload. This fetus attempts to compensate by increasing urine output. Excessive urine production then distends their amniotic sac (polyhydramnios). At birth the recipient may appear large, swollen and red. Accumulation of amniotic fluid may cause uterine contractions, with pressure on the uterus and cervix triggering premature labour or precipitating preterm premature rupture of the membranes.

TTTS usually becomes clinically evident during the mid-trimester (16–21 weeks). Signs may include a rapid and marked increase in a mother’s abdominal girth, due to the recipient’s expanded amniotic fluid compartment. However, subclinical TTTS is often identified on ultrasound earlier in the pregnancy. The onset may be slow (over weeks) or acute and catastrophic. The diagnosis of TTTS is based on strict ultrasonographic criteria [7], of which the most widely used is the Quintero staging system [8]. The possibility of TTTS is one of the reasons behind frequent antenatal ultrasound monitoring of monochorionic pregnancies.

### Natural history

Mortality from severe untreated TTTS is extremely high, with rates between 70 and 100% reported [9, 10]. Deaths occurring in utero are usually attributable to fetal cardiac failure. Without treatment, pre-viable or extremely preterm births contribute to high perinatal mortality. Of twins that are liveborn, a significant proportion suffer from postnatal complications of TTTS, including heart and kidney dysfunction and complications of polycythaemia and anaemia [10, 11].

### Selective Fetoscopic laser photocoagulation of placental anastomoses (SFLP or “laser surgery”)

In 1983, Dr. Julian De Lia and colleagues began developing a novel therapy for TTTS. Working initially with sheep (which have a naturally high rate of identical twinning), and subsequently humans, they pioneered the procedure of fetoscopic placental laser surgery [12]. Quintero and colleagues subsequently demonstrated superiority of a selective over a non-selective approach to obliteration of vessels at the vascular equator [13]. In a further refinement, points of coagulation were joined by a line of coagulation across the vascular equator (Solomon technique). This technique significantly reduced post-laser complications of recurrent TTTS and twin anaemia polycythaemia sequence (TAPS, an atypical chronic form of TTTS) [14].

Prior to the development of laser surgery, the only management options for TTTS were palliative, including amnioreduction (drainage of excess amniotic fluid to relieve uterine pressure) or septostomy (creating a hole in the inter-fetal membrane allowing equalisation of fluid). In cases of fetal malformation, selective termination of one fetus was employed in the hope of improving outcomes for the less affected fetus/es.

Unlike amnioreduction and septostomy, SFLP offers a cure for the underlying pathological process. The procedure identifies and physically disrupts the anastomosing vessels, thereby preventing transfusion of blood from donor to recipient [15]. Initially performed using an open approach with laparotomy, SFLP now usually uses a minimally invasive laparoscopic technique. Maternal physical recovery from the procedure is prompt. Rarely, a second (repeat) laser procedure is required should TTTS recur or post-laser TAPS develop, either as a result of a “missed” vessel/s, or as a novel episode [16]. Surgical failure may occur in up to 18% of procedures [7] and preterm labour and preterm premature rupture of membranes also contribute to post-operative perinatal morbidity and mortality.

In 2004, a randomised controlled trial comparing laser surgery with (then-standard) serial amnioreductions had to be discontinued early when interim analysis demonstrated clear superiority of laser in terms of survival and survival without major disability [17]. SFLP is now first line treatment for all but the mildest cases of TTTS, and a randomized controlled trial is currently underway, examining the role of SFLP in Stage 1 TTTS [18].

### Australian experience with laser surgery, and the Victorian fetal therapy service (VFTS)

The first fetal laser surgery for TTTS in Australia was performed at the Mater Hospital in Brisbane in 2002 [19]. Today, SFLP is offered in four Australian states: Queensland, New South Wales, Western Australia and Victoria. The Victorian Fetal Therapy Service (VFTS) is a three-centre collaboration between Monash Health, Mercy Hospital for Women (MHW) and The Royal Women’s Hospital (RWH), with surgery conducted at Monash Health. Reported outcomes have been consistent with international experience [14, 17, 20, 21], with 68% overall infant survival, and survival of one or more twin/s in 86% of gestations treated.

Regrettably, in Melbourne to date there has been no formal system for routine neurodevelopmental follow-up of surviving children. Although at elevated risk of neurodevelopmental disability, follow-up has been at the discretion of the clinicians involved in the children’s postnatal care. Lack of consistency of follow-up may have resulted in missed opportunities for early detection of developmental difficulties, and valuable information has not been collected.

### Neurodevelopmental outcomes following TTTS

Prevalence of severe neurodevelopmental abnormalities among monochorionic twins who *did not* suffer from TTTS is between 4 and 8% [22]. Survivors of TTTS have been demonstrated to be at further increased risk of adverse neurodevelopmental outcomes. Prior to widespread adoption of SFLP, rates of neurological disability documented among TTTS survivors ranged between 17% [23] and 42% [24].

Van Klink and colleagues [25] summarised 13 studies from 1999 to 2016, reporting neurodevelopmental outcomes following laser surgery. Observed rates of cerebral palsy ranged between 3 and 12%, and rates of neurodevelopmental impairment (cerebral palsy, severe cognitive and/or motor delay (< 2 SD), blindness and/or deafness) were 4–18%. Table 1 summarises an additional 4 studies. Lower prevalence of disability has been identified following briefer periods of follow-up (6 months-2 years) and using less structured review procedures, and higher prevalence with longer duration of follow-up and more rigorous evaluation.

**Table 1:** Developmental Outcomes following laser for TTTS (subset of published studies)

Author and year	Number and age of participants	Measures	Outcomes
Campos, D., et al., (2016) [35]	*N* = 33 monochorionic diamniotic twins (post-laser), and *N* = 22 term singletons Birth to 12 months, corrected for prematurity	Bayley Scales of Infant Development, Clinical examination of TTTS group. Two assessments, in first and second 6 months of life.	Cerebral palsy in 18%, strabismus in 9%, microcephaly in 3% of TTTS group. Significant difference between groups in prevalence of cognitive and fine motor deficits apparent by 6 months (greater risk in TTTS group); by 12 months, significantly greater prevalence of deficits in all domains for TTTS group Comparative results at second assessment not provided (table of first assessment repeated in error). Donors 7 times increased risk of adverse outcomes c.f. recipients; donor status and low socioeconomic status, and cardiorespiratory disease were associated with poorer expressive communication & fine motor skills respectively
Müllers, S. et al., (2015) [36]	*N* = 106 (post-laser). Median age 4 years (range 6 mo – 7 yrs) (correction for prematurity not specified)	Individual correspondence and paediatric evaluation (details not specified)	Ongoing neurodevelopmental concerns in 14% (speech and language concerns *n* = 7, behavioural concerns *n* = 2, mild motor delay n = 2, mild cerebral palsy n = 2, major cerebral palsy n = 2)
Tosello, B. et al., (2014) [29].	*N* = 35 (post-laser). Median age 37 months, mean 30 mo (range 4 mo - 5 yrs)	Neurological assessment at discharge from maternity hospital. Ages and Stages Questionnaire (ASQ) at up to 5 years	As neonates, ≈7% neurologically abnormal (≈93% normal). At follow-up, ≈31% abnormal based on ASQ (≈69% normal), ≈6% severely neurologically abnormal (cerebral palsy). Of children found to be abnormal at follow-up, 45% had not been detected on routine medical review. Donor status and birth < 32 weeks significantly associated with adverse neurosensory outcome as neonates. No correlations at follow-up between outcome and donor status, severity of TTTS or other variables (but small numbers)
Sago, H. et al., (2010) [37]	*N* = 275 (post-laser). Age 6 months (correction for prematurity not specified)	Review of cerebral imaging & clinical assessment by paediatrician (details not specified)	Major neurological disability in ≈5% (severe IVH, cystic PVL, CP, hydrocephalus, ventriculomegaly, or multiple infarcts)

Characterisations of neurodevelopmental outcomes in the literature have been problematic and managed inconsistently, for several reasons. First, developmental status is often reported as a categorical variable (“impaired” vs “unimpaired”), whereas a more conceptually sound framework sees neurodevelopment on a continuum of ability [26]. Further, neurodevelopment is not a single entity, but is conceptualised as comprising a number of domains, any one of which may be impaired either in isolation or in combination. Assigning a child to an “outcome” implies that this outcome is fixed, and ignores that change is fundamental to the construct of neurodevelopment.

Thus far, reports of measures of social or emotional development, or academic achievement, have been lacking. Findings regarding language have relied on subscales of global measures of intelligence, rather than specific measures of speech and language. This is a considerable oversight, given the documented high prevalence of language disorders among children of multiple birth [27, 28]. Consideration of lesser degrees of neurodevelopmental impairment has also been overlooked. Severe outcomes such as cerebral palsy have been reported, but prevalence estimates of relatively minor morbidities (such as specific learning impairments) that may nonetheless have significant impacts on the lives of survivors and their families are not available. The most common approach in reporting of outcomes has been a three-tier categorisation of “Normal”, “Mild impairment”, and “Severe impairment”. Children involved in this study will be similarly grouped allowing comparison with previous reports, but due to the limitations of this approach, alternative outcomes, described in the methods, will also be reported.

### Study aims

The study will assess child survivors of TTTS-affected multiple pregnancies managed by fetal laser surgery in Victoria for the presence of neurodevelopmental disabilities, and will establish a database of obstetric, neonatal and paediatric data relating to this disorder. The database will serve as a model for future, prospective research involving children at risk of developmental disabilities.

## Method

Approval for the study was obtained from the Human Research Ethics Committees (HRECs) of the Royal Children’s Hospital (reference 34269D) Royal Children’s Hospital (reference 34269D), Monash Health (reference RES-17-0000-149X), Mercy Hospital for Women (reference R15/24) and The Royal Women’s Hospital (reference HREC/15/RCHM/37). Consent is informed opt-in by parent or legal guardian (either written or verbal, which in the latter case must be documented by a researcher). A separate consent form will be completed for each participating child. Consent forms will be kept in a locked cabinet on MCRI premises, and scanned into the database.

### Study design

The proposed study is a retrospective cohort study assessing neurodevelopmental outcomes for survivors of TTTS managed with laser photocoagulation in Melbourne, Australia, from 2006 to 2015 inclusive.

### Study setting

The study will be coordinated through the department of Developmental Disability and Rehabilitation Research, at the Murdoch Children’s Research Institute (MCRI). Monash Health was the location of all laser surgery. Follow-up assessments will be conducted at either of the two tertiary children’s hospitals in the Australian state of Victoria, or in the family home (depending on family preference), with reimbursement of travel expenses and hospital parking fees. The relevant departments at The Royal Children’s Hospital and Monash Health are Neurodevelopment and Disability and Monash Paediatric Rehabilitation Service, respectively. The research team will consist of a doctor, psychologist, speech therapist, physiotherapist or occupational therapist and a study coordinator. For each participating family, appointments will be scheduled to take place on a single day.

### Participants

The study includes surviving twin (or triplet) child participants, aged 24 months or more (corrected for prematurity), and their parents or carers who will report on the abilities and behaviour of their child/ren. Where survival is not documented or is not known, the family will be approached in a sensitive manner to ascertain eligibility.

#### Number of participants

Approximately 100 procedures have been undertaken within the specified range. Overall survival has been reported at 68% [20]. Assuming twin gestations, approximately 136 children are eligible for the study (100 × 2 × 0.68).

### Procedure: Recruitment strategy

**Fig. 1:**
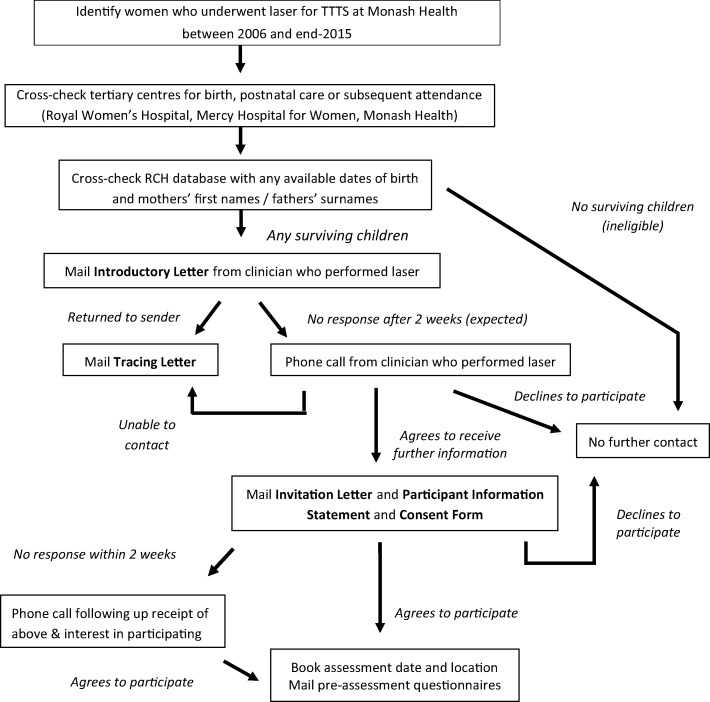
Recruitment strategy

Recruitment strategy is outlined in Fig. 1 below.

#### Loss to follow-up

Previous similar studies have identified a loss to follow up proportion of up to 20% at a median age of 37 months [29]. As the present study involves a greater time since the procedure (up to 11 years, in the case of procedures performed in 2006), the proportion lost to follow up is likely to be higher.

As far as possible, characteristics of potential participants lost to follow-up will be compared with those able to be followed up, to identify whether significant differences (in terms of characteristics such as severity of TTTS) exist between groups. Previous studies have not found significant differences between these groups.

### Measures: Core components

#### Pre-assessment completion of standardised screening questionnaires

For each child, parents/carers will complete one or more of the Ages and Stages Questionnaire 3rd edition (ASQ-3), the Infant Toddler Social Emotional Assessment (ITSEA) and the Childhood Behaviour Checklist (CBCL) (see Table 2 and Additional file 1 for further information). These assess social, emotional and behavioural skills and general development.

**Table 2:** Schedule of Standardised developmental assessments by age

	Age of participant		
	24 – 36mo	3y – 6y 11mo	7y +
General Cognition (administered by paediatric psychologist)			
Cognitive Scale from Bayley Scales of Infant and Toddler Development 3rd Ed (Bayley–III)***	✔		
Wechsler Preschool and Primary Scale of Intelligence 4th Ed (WPPSI-IV) Core subtests (30–60 min)		✔	
Wechsler Intelligence Scale for Children 5th Ed (WISC-V) Core subtests (60 mins)			✔
Motor Skills (administered by paediatric occupational therapist or physiotherapist)			
Fine & Gross Motor Scales (Bayley-III)***	✔		
Movement Assessment Battery for Children 2nd Ed (MABC-2)* (20–40 min)		✔	✔
Gross Motor Function Classification Score (GMFCS-E&R) if applicable (5–20 min)	✔	✔	✔
Manual Ability Classification Score (MACS) if applicable	✔	✔	✔
Language and Communication (administered by paediatric speech therapist)			
Receptive & Expressive Scales (Bayley-III)***	✔		
Clinical Evaluation of Language Fundamentals Preschool – 2 (CELF-P2)* Core subtests (30–60 min)		✔	
Clinical Evaluation of Language Fundamentals (CELF-IV) Core subtests (30–60 min)			✔
Communication Function Classification System (CFCS) if applicable	✔	✔	✔
Social/Emotional / Behavioural skills and General Development (parent report questionnaire)			
Infant Toddler Social Emotional Assessment (ITSEA) (25–30 min)	✔		
Child Behavior Checklist (CBCL) (15 mins)		✔	✔
Ages and Stages Questionnaire (ASQ-3)** (10–15 min)	✔	✔	
Academic Achievement (administered by paediatric psychologist)			
Wide Range Achievement Test 4th Ed (WRAT-4) (select subtests) (15–25 min)			✔

*Some younger children may need to be assessed with the previous age group’s instrument; ** Children ≤5 years; ***45–60 min total

#### Medical assessment

Each child will undergo a structured assessment by a developmental paediatrician or paediatric neurologist, including sociodemographics, measurements of height, weight and head circumference, and developmental and neurological status. Clinical history will be obtained from the parent/guardian. Children may be referred for further evaluation (e.g. audiology, blood tests, imaging) if indicated.

Should assessment identify concerns which were previously unknown, parents’ permission will be sought to notify the child’s usual doctor of the findings. Where appropriate, children may be referred for further clinical assessment, opinion or ongoing management.

#### Standardised developmental assessments

Assessments will be tailored to each child’s age, as listed in Table 2. Each instrument is well validated for paediatric use, and normative data are available for comparison with the study group (refer to Additional file 1 for further information). Scores will be calculated using both chronological age, and age corrected for prematurity [30].

### Measures: Optional component

#### Medical information from hospital of birth and subsequent health care providers

Parents will be asked whether they consent to researchers seeking medical information from their child/ren’s hospital of birth, and from subsequent providers of health care.

Seeking background information is important because risk factors which may be associated with neurodevelopmental outcomes may not be known to the parent (such as severity of TTTS, extent of resuscitation required at birth or results of neonatal imaging). Where results of previous developmental assessments (particularly formal IQ tests) are available and are considered reliable and current (undertaken within the past 2 years), such assessments will not be repeated.

### Outcome measures

#### Outcome by overall neurological status

As has been the case with previous international reports, a three-tiered outcome measure for each child will be allocated (see Table 3). Definitions of these categories will be consistent with previously published reports [31, 32].

**Table 3:** Operational definitions of Overall Neurodevelopmental Outcome Groups

Group 1: Unimpaired	Group 2: Mild neurological and/or developmental impairment	Group 3: Severe neurological and/or developmental impairment
NO neurological findings on history / examination, AND no functional impairment	ANY neurological findings on history/examination which are objectively mild or moderate AND which DO NOT result in severe functional impairment *Examples: Strabismus (squint), Mild talipes (club foot), Mild cerebral palsy (GMFCS I-II)*	ANY neurological findings on history/examination which are objectively moderate or severe AND which result in severe functional impairment *Examples: Moderate-Severe cerebral palsy (GMFCS 3–5), Severe visual impairment*
NO neurodevelopmental delay or disability in any domain (either on clinical assessment or according to standardised measures) AND no functional impairment	ANY neurodevelopmental delay or disability (in one or more domain/s, either on clinical assessment or according to standardised measures), which is objectively mild or moderate, AND which DOES NOT result in severe functional impairment *Examples: Mild intellectual disability (IQ 50-70), Mild Autism Spectrum Disorder, Mild Isolated Speech Delay*	ANY neurodevelopmental delay or disability (in one or more domain/s, (either on clinical assessment or according to standardised measures), which is objectively moderate or severe, AND which results in severe functional impairment *Examples: Moderate or Profound intellectual disability (IQ 35–49, or < 30), Moderate or severe Autism Spectrum disorder*

In cases of uncertainty (e.g. moderate gross motor disability (Group 2) but severe functional impairment (Group 3), or severe neurological deficit (Group 3) but moderate functional impairment (Group 2), participants will be classified according to degree of functional impairment.

Medical and allied health clinicians will provide their opinion on each child’s neurodevelopmental status, and assignment of overall neurodevelopmental outcome will be achieved by consensus of a panel of clinicians.

#### Outcome by specific neurodevelopmental diagnosis, developmental domains, and academic achievement

Outcomes will also be categorised by clinical entity (e.g. percentage of children affected by cerebral palsy, including pattern and severity; percentage of children affected by autism, mild or severe). Some children are likely to have more than one diagnosis. Outcomes according to impairment in each of the developmental domains will also be reported, as will results of academic testing for children old enough to participate.

#### Feedback following assessment

Approximately a month following the assessment, parents will receive a brief (2 page) written report by mail, summarising their child/ren’s assessment results. This will be followed by a phone call from a member of the research team within two weeks (parents may opt out of this contact by leaving a phone message or email). In keeping with standard practice among similar research studies, individual children’s numerical assessment scores will not be released unless parents explicitly request this information.

#### Data management

Contact information of potential participants will be recorded on password-protected spreadsheets.

Clinical notes and raw and standardised assessment scores of study participants will be captured by REDCap electronic data capture tools [33] hosted by MCRI. This software allows authorised researchers at distant sites to access the database and add information. With parents’ permission, previous medical reports and images will be able to be attached to child participants’ files. Raw data (including clinical notes, questionnaires and assessment forms) will be retained for the periods prescribed in the MCRI Research Data Storage, Retention & Disposal Policy & Procedure (MCRI4002) (at least until participants reach the age of 25 years).

#### Data analysis

The REDCap database allows information to be transferred to statistical software for analysis. Analysis will be performed on de-identified data. Descriptive statistics will include means and percentages of participants with given outcomes. Data analysis will include Pearson’s χ^2^ and Fisher’s exact tests (when *n* < 5), and 2-factor analysis of variance (ANOVA). Scale variables (such as gestation at birth) will be examined for distribution of scores, with normality testing using Kolmogorov-Smirnov and Shapiro-Wilk tests. Univariate analysis will identify factors associated with outcomes of interest (such as survival without disability), and will include calculations of Odds Ratios. Structured equation modelling will be used to explore the causal pathways and interactions between possible causal factors of adverse neurodevelopmental outcomes. When considering statistical associations and comparisons, the *p* value will be used to assess the strength of association.

## Discussion: Ethical considerations

### Sensitivity to bereavement

As a high-risk population, it is anticipated that a significant proportion of parents will be bereaved of one or more children. It is for this reason that birth and postnatal records will be reviewed prior to recruitment. Although no parent who lost both twins or all triplets will knowingly be invited to participate, it is theoretically possible that a parent may receive written study information before the researchers become aware of the bereavement. For this reason, the Introductory Letter from the laser clinician is non-specific. The Participant Information Statement includes the following text:

“Please note: We have tried very hard to avoid sending project information to parents whose children have both / all passed away. If you are in this situation and you have received this by mistake, please accept our sincerest apologies and condolences, and kindly disregard the information.”

### Potential for psychological discomfort among parent/guardian participants

Participation may be associated with some psychological discomfort for parents, as the study recalls a time of uncertainty for their children’s survival. The risk is considered low. Special counselling due to participation in this study itself is not necessary, however the population is recognised to be at significant risk of mental health and adjustment difficulties [34]. Should a parent show signs of distress, contact information for relevant support services will be offered, such as PaNDA (post- and antenatal depression association), or AMBA (the Australian Multiple Birth Association). The Participant Information also includes contact details for relevant services.

### Potential biases and limitations to feedback

A problem which is unique to the twin / triplet situation is the possibility of parents or clinicians comparing a child’s development with his/her co-multiple/s, rather than with the wider population of children (as is more appropriate). This can lead to false inflation of differences which are, in fact, very minor. The ability levels of children within intact sets will not be compared (unless parents explicitly request this information). Comparisons with “children in general” may be made, as is standard practice when discussing assessment findings. This will minimise risk of distress to children due to participation in the study.

Test scores will not be released to participating families. As an example of potential damage caused by releasing scores in this context, one child may receive a full-scale IQ score of 92. His twin may receive a score of 89. The difference between these test scores is clinically meaningless (and is likely to reflect an approximately equal “true” score), however this can be a difficult concept to convey to a lay audience. If these numbers were provided to the parents, they may interpret them to mean that the first twin was “smarter” than the second. This could influence the way in which they subsequently interact with their children, to the detriment of one or both.

The exception to the default position (non-provision of scores) is when a child’s assessment indicates an ability level likely to result in significant functional difficulties that could benefit from interventions or supports. In these instances, scores will be conveyed to parents. For example, a child whose full-scale IQ falls below 70 is likely to have trouble functioning in a standard classroom without modifications. If the children within a set operate at substantially different levels, it may be unavoidable that a distinction between their levels of function will be drawn, but this is likely to already be evident to their parents or guardians and will not be deliberately emphasised.

### Implications of study findings

The study will fill a significant knowledge gap regarding outcomes for Victorian children with TTTS undergoing SFLP, and contribute to international knowledge about prevalence and severity of adverse neurodevelopmental outcomes. It has implications for service delivery, as it may help clarify whether universal follow-up of survivors is warranted, or whether a subgroup of children should be assessed at routine intervals to allow timely identification of neurodevelopmental problems. In addition, the study will inform future research into factors on the pathway to neurodevelopmental disability for children treated with SFLP.

## Additional file

Additional file 1: (“Psychometric properties of instruments used in the Twin-to-twin Transfusion Syndrome Developmental Follow-Up Study”). (DOCX 24 kb)

## Abbreviations

AMBA: Australian Multiple Birth Association
CP: Cerebral palsy- a persistent but not unchanging disorder of movement and posture due to a defect or lesion of the developing brain
HREC: Human Research Ethics Committee
MCRI: Murdoch Children’s Research Institute
PaNDA: Post- and antenatal depression association
SFLP: Selective Fetoscopic Laser Photocoagulation of placental anastomoses
TTTS: Twin-to-twin transfusion syndrome
VFTS: Victorian Fetal Therapy Service
