# Supplementary material for: Twin-to-twin transfusion syndrome neurodevelopmental follow-up study (neurodevelopmental outcomes for children whose twin-to-twin transfusion syndrome was treated with placental laser photocoagulation)
Source: BMC Pediatr. 2018 Aug 1;18:256. doi: 10.1186/s12887-018-1230-8 (PMC6090765; doi:10.1186/s12887-018-1230-8)
Supplement: Supplementary file 1 — (“Psychometric properties of instruments used in the Twin-to-twin Transfusion Syndrome Developmental Follow-Up Study”). (DOCX 24 kb) [file 12887_2018_1230_MOESM1_ESM.docx]

Additional File 1:

**Psychometric properties of instruments used in the Twin-to-twin Transfusion Syndrome Developmental Follow-Up Study**

# Bayley Scales of Infant and Toddler Development 3^rd^ Ed (BSID-III)

The BSID-III are norm-reference instruments for assessing development of children aged from 1 to 42 months. The instrument takes 45-60 minutes to administer. Results are reported on cognitive, language and motor scales. Reliability and validity are reportedly high. The mental and motor scales have high correlation coefficients (.83 and .77) for test/retest reliability. The Bayley Scales have been used in previous research involving survivors of TTTS [1, 2]. Local data will be used for comparison ([3-5].

# Wechsler Preschool and Primary Scale of Intelligence 4^th^ Edition (WPPSI-IV)

The WPPSI-IV takes between 30-60 minutes to complete. It yields scores in four specified cognitive domains: Verbal Comprehension, Perceptual Reasoning, Processing Speed and Working Memory. Reliability and validity are within acceptable limits [6]. Exclusionary factors include (i.e., the test is unsuitable for assessment in the following circumstances) speech and language disabilities, upper limb movement difficulties, and physical or medical conditions which may adversely affect test performance. Under these conditions, as many of the core subtests as possible will be administered, with the caveat that interpretation of results will require particular caution.

# Wechsler Intelligence Scale for Children 5^th^ Ed (WISC-V)

The WISC-V is a measure of overall intellectual ability for children aged 6-16 years. It takes 1 hour to complete core tests. The instrument yields a full-scale IQ score (FSIQ) and primary and ancillary index scores. It provides a Full Scale IQ (FSIQ) score measuring general intellectual ability. It has satisfactory psychometric properties [7] and studies support its use in broad contexts, including with children with learning disabilities [8]. A previous version of this instrument (the WISC-IV) was used in similar research involving survivors of TTTS [9].

# Movement Assessment Battery for Children 2^nd^ Ed (MABC-2)

The MABC-2 [10] is a standardised, norm-referenced instrument which identifies and describes movement impairments in children. It takes between 20 and 40 minutes to complete. It has been demonstrated to be a reliable and valid instrument for identification of movement disorders in children aged 3-5 years [11]. In older children (6-12 years), the reliability of the MABC-2 has been found to be excellent (intra-class correlation coefficient 0.97, internal consistency Cronbach’s alpha

0.9)[12]

# Gross Motor Function Classification Score – Expanded & Revised (GMFCS-E&R)

This well-validated instrument was developed in 1997 and extended in 2008, as a classification system for the movement abilities of children and youth with cerebral palsy [13]. It takes between 5 minutes (familiar clinician) and 15-20 minutes (unfamiliar clinician) to complete. Five functional levels were clearly defined and illustrated, describing a person’s current motor function, It includes the use of mobility aids, and can be used from 2 to 18 years.

# Manual Ability Classification Score (MACS)

This instrument classifies the typical daily use of hands by children with cerebral palsy, on 5 levels. It has been demonstrated to have good reliability and validity, with excellent agreement between raters. [14]

# Clinical Evaluation of Language Fundamentals Preschool – 2 (CELF-P2) and CELF-4

The CELF-P2 and CELF-4 take between 30-60 minutes to complete. They each yield a core language score, receptive and expressive language index scores, expressive language, language content, and language structure index scores; standard scores, percentile ranks, age equivalents, and growth scores. Test-retest stability has been shown to be high, and Cronbach’s alphas are within acceptable limits. Validity studies have included children with language disorders, mild autism, hearing impairments and intellectual disability [15].

# Communication Function Classification System (CFCS)

This instrument was developed to classify the ability of a person with cerebral palsy to communicate with a partner (including individuals who use augmented communication methods). Similar to the GMFCS and MACS, it is quickly completed on a five level scale, and has very good test-retest reliability, and good inter-rater reliability. It can be used from 2 years of age.[16]

# Infant Toddler Social Emotional Assessment (ITSEA)

This instrument assesses emerging social and emotional development in young children aged between 12 and 36 months. It takes 25-30 minutes to administer, and is completed by a parent. It provides detailed analysis of 17 subscales within four domains (externalising and internalising problems, dysregulation and competence). The instrument has been validated and was shown to have robust psychometric properties [17]. The ITSEA will be provided to parents of children aged under 36 months (corrected age). For children over 36 months (corrected age), the Child Behaviour Checklist will be completed.

# Child Behavior Checklist (CBCL)

The CBCL is an assessment tool which identifies emotional and behavioural difficulties in children and adolescents, and is completed by a parent or carer. The instrument takes 15 minutes to complete, with responses measured on Likert scales. It is available either as a paper version or online. Versions are available both for younger children (1 ½ to 5 years) and older children (6-18 years). The CBCL will be provided to parents of children aged over 36 months (corrected age). Results yielded describe children in terms of percentile rankings for particular clusters of difficulty (Internalising, Externalising problems and subsets of each, Sleep problems and Overall problems) Validity and reliability are well established [18].

# Ages and Stages Questionnaire 3^rd^ edition (ASQ-3)

This standardised instrument includes language, personal-social, fine and gross motor, and problem solving skills, and takes between 10-15 minutes to complete. It has been demonstrated to have satisfactory psychometric properties (sensitivity 75%, specificity 81%), including in evaluation of preterm children [19]. The ASQ-3 will be completed by parents of children aged ≤5 years. It has good negative predictive value, and correlates well with developmental scales. [20]. There is a precedent for using this instrument in similar research [21].

# Wide Range Achievement Test 4^th^ Ed (WRAT-4)

The WRAT-4 takes 15-25 minutes to complete. It provides an assessment of basic academic skills of reading, spelling, and mathematical computation, and may be used from 5 years. High reliability and validity were demonstrated using a representative sample of over 3000 individuals, and comparison with special groups of individuals, and correlations with other widely used achievement and cognitive ability measures [22].

1. Gray PH, Poulsen L, Gilshenan K, Soong B, Cincotta RB, Gardener G: **Neurodevelopmental outcome and risk factors for disability for twin-twin transfusion syndrome treated with laser surgery**. *American journal of obstetrics and gynecology* 2011, **204**(2):159.e151-156.
2. Lopriore E, Ortibus E, Acosta-Rojas R, Le Cessie S, Middeldorp JM, Oepkes D, Gratacos E, Vandenbussche FP, Deprest J, Walther FJ *et al*: **Risk factors for neurodevelopment impairment in twin-twin transfusion syndrome treated with fetoscopic laser surgery**. *Obstetrics and Gynecology* 2009, **113**:361-366.
3. Anderson PJ, De Luca CR, Hutchinson E, Roberts G, Doyle LW, the Victorian Infant

Collaborative Group: **Underestimation of Developmental Delay by the New Bayley-III Scale**. *Arch Pediatr Adolesc Med* 2010, **164**(4):352-356.

1. Spittle AJ, Spencer-Smith MM, Eeles AL, Lee KJ, Lorefice LE, Anderson PJ, Doyle LW: **Does the Bayley-III Motor Scale at 2 years predict motor outcome at 4 years in very preterm children?** *Developmental Medicine & Child Neurology* 2013, **55**(5):448-452.
2. Hutchinson EA, De Luca CR, Doyle LW, Roberts G, Anderson PJ, Group ftVICS: **School-age Outcomes of Extremely Preterm or Extremely Low Birth Weight Children**. *Pediatrics* 2013, **131**(4):e1053-e1061.
3. Canivez GJ: **Review of the Wechsler Preschool and Primary Scale of Intelligence- Fourth Edition**. In*.* Charleston, IL: BUROS Center for Testing; 2010.
4. Weiss LG, Keith TZ, Zhu J, Chen H: **WISC-IV and Clinical Validation of the Four- and FiveFactor Interpretative Approaches**. *Journal of Psychoeducational Assessment* 2013, **31**(2):114-131.
5. Styck KM, Watkins MW: **Structural Validity of the WISC-IV for Students With Learning Disabilities**. *Journal of Learning Disabilities* 2014.
6. Salomon LJ, Ortqvist L, Aegerter P, Bussieres L, Staracci S, Stirnemann JJ, Essaoui M, Bernard JP, Ville Y: **Long-term developmental follow-up of infants who participated in a randomized clinical trial of amniocentesis vs laser photocoagulation for the treatment of twin-to-twin transfusion syndrome**. *American journal of obstetrics and gynecology* 2010, **203**(5):444.
7. Henderson SE, Sugden DA, Barnett AL: **Movement Assessment Battery for Children–2.** In*.* London: Harcourt Assessment; 2007.
8. Ellinoudis T, Evaggelinou C, Kourtessis T, Konstantinidou Z, Venetsanou F, Kambas A: **Reliability and validity of age band 1 of the Movement Assessment Battery for Childrensecond edition.** *Research in Developmental Disabilities* 2011, **32**(3):1046-1051.
9. Wuang YP, Su JH, Su CY: **Reliability and responsiveness of the Movement Assessment Battery for Children – second edition test in children with developmental coordination disorder**. *Child Neurology* 2012, **54**(2):160-165.
10. Palisano RJ, Rosenbaum P, Bartlett D, Livingston MH: **Content validity of the expanded and revised Gross Motor Function Classification System**. *Developmental Medicine & Child Neurology* 2008, **50**(10):744-750.
11. Eliasson A, Krumlinde-Sundholm L, Rösblad B, Beckung E, Arner M, Öhrvall A, Rosenbaum P: **The Manual Ability Classification System (MACS) for children with cerebral palsy: Scale development and evidence of validity and reliability**. *Developmental Medicine & Child Neurology* 2006, **48**(7):549-554.
12. **CELF-4 Technical Report**. In*.*: Pearson Education; 2008.
13. Hidecker MJC, Paneth N, Rosenbaum PL, Kent RD, Lillie J, Eulenberg JB, Chester JK, Johnson B, Michalsen L, Evatt M *et al*: **Developing and validating the Communication Function Classification System for individuals with cerebral palsy**. *Developmental Medicine & Child Neurology* 2011, **53**(8):704-710.
14. Carter AS, Briggs-Gowan MJ, Jones SM, Little TD: **The Infant-Toddler Social and Emotional Assessment (ITSEA): factor structure, reliability, and validity**. *Journal of Abnormal Child Psychology* 2003, **31**(5):495-514.
15. Achenbach TM, Rescorla LA: **Manual for ASEBA school-age forms & profiles**. In*.* Burlington: University of Vermont, Research Center for Children, Youth, & Families; 2001.
16. Schonhaut L, Armijo I, Schönstedt M, Alvarez J, Cordero M: **Validity of the Ages and Stages Questionnaires in Term and Preterm Infants**. *Pediatrics* 2013, **131**(5):e1468-e1474.
17. Skellern CY, Rogers Y, O'Callaghan MJ: **A parent-completed developmental questionnaire:**

**follow up of ex-premature infants**. *Journal of Paediatrics and Child Health* 2001, **37**(2):125129.

1. Tosello B, Blanc J, Haumonté J, D’Ercole C, Gire C: **Short and medium-term outcomes of liveborn twins after fetoscopic laser therapy for twin-twin transfusion syndrome**. *Journal of Perinatal Medicine* 2014, **42**(1):99-105.
2. Wilkinson GS, Robertson GJ: **Wide Range Achievement Test 4 professional manual**. In*.* Lutz, FL: Psychological Assessment Resources 2006.
